# Subchronic exposure to 1,2-naphthoquinone induces adipose tissue inflammation and changes the energy homeostasis of mice, partially due to TNFR1 and TLR4

**DOI:** 10.1016/j.toxrep.2023.06.002

**Published:** 2023-06-15

**Authors:** Clílton Kraüss de Oliveira Ferreira, Clara Machado Campolim, Olívia Pizetta Zordão, Fernando Moreira Simabuco, Chadi Pellegrini Anaruma, Rodrigo Martins Pereira, Vitor Ferreira Boico, Luiz Guilherme Salvino, Maíra Maftoum Costa, Nathalia Quintero Ruiz, Leandro Pereira de Moura, Mario Jose Abdalla Saad, Soraia Katia Pereira Costa, Young-Bum Kim, Patricia Oliveira Prada

**Affiliations:** a Faculty of Applied Sciences, State University of Campinas, Limeira, SP, Brazil; b Department of Internal Medicine, Faculty of Medical Science, State University of Campinas, Campinas, SP, Brazil; c Department of Physical Education, Institute of Biosciences - São Paulo State University, Rio Claro, SP, Brazil; d Department of Pharmacology, Institute of Biomedical Sciences, University of São Paulo, São Paulo, SP, Brazil; e Department of Medicine, Beth Israel Deaconess Medical Center, Harvard Medical School, Boston, MA, USA; f Max-Planck Institute for Metabolism Research, Köln, Germany

**Keywords:** Air pollution, Diesel, PM_2.5_, 1, 2-naphthoquinone, Energy balance, Inflammation, White adipose tissue, Macrophages

## Abstract

Air pollution affects energy homeostasis detrimentally. Yet, knowledge of how each isolated pollutant can impact energy metabolism remains incomplete. The present study was designed to investigate the distinct effects of 1,2-naphthoquinone (1,2-NQ) on energy metabolism since this pollutant increases at the same rate as diesel combustion. In particular, we aimed to determine in vivo effects of subchronic exposure to 1,2-NQ on metabolic and inflammatory parameters of wild-type mice (WT) and to explore the involvement of tumor necrosis factor receptor 1 (TNFR1) and toll-like receptor 4 (TLR4) in this process. Males WT, TNFR1KO, and TLR4KO mice at eight weeks of age received 1,2-NQ or vehicle via nebulization five days a week for 17 weeks. In WT mice, 1,2-NQ slightly decreased the body mass compared to vehicle-WT. This effect was likely due to a mild food intake reduction and increased energy expenditure (EE) observed after six weeks of exposure. After nine weeks of exposure, we observed higher fasting blood glucose and impaired glucose tolerance, whereas insulin sensitivity was slightly improved compared to vehicle-WT. After 17 weeks of 1,2-NQ exposure, WT mice displayed an increased percentage of M1 and a decreased (p = 0.057) percentage of M2 macrophages in adipose tissue. The deletion of TNFR1 and TLR4 abolished most of the metabolic impacts caused by 1,2-NQ exposure, except for the EE and insulin sensitivity, which remained high in these mice under 1,2-NQ exposure. Our study demonstrates for the first time that subchronic exposure to 1,2-NQ affects energy metabolism in vivo. Although 1,2-NQ increased EE and slightly reduced feeding and body mass, the WT mice displayed higher inflammation in adipose tissue and impaired fasting blood glucose and glucose tolerance. Thus, in vivo subchronic exposure to 1,2-NQ is harmful, and TNFR1 and TLR4 are partially involved in these outcomes.

## Highlights

• Air pollution affects energy homeostasis detrimentally.
• Knowledge of how each isolated pollutant can impact energy metabolism remains unclear.
• Specific effects of 1,2-naphthoquinone on energy metabolism are unknown.
• 1,2-naphthoquinone increases at the same rate as diesel combustion.

## Graphical Abstract

**Figure:**
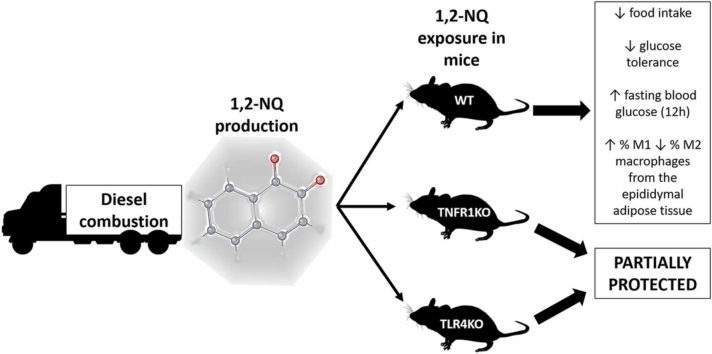

## 1 Introduction

Air pollution emerged as a significant health risk factor due to increased industrialization and urban traffic in many countries. Also, there is a recent estimation of excess deaths in Europe and worldwide attributable to air pollution using novel exposure-response functions [33], [32]. The World Health Organization (WHO) targets air pollution as up to 15 μg/m^3^/day even though the top ten polluted cities emit an average of 80–110 μg/m^3^ [65]. Particulate matter (PM) is the primary harmful fraction of air pollution impairing health. The composition and size of PM depends on emission sources, including road dust and a substantial amount of diesel exhaust particles coming from urban traffic [3], [61]. Chronic exposure to the ambient fine particulate matter with aerodynamic diameter ≤ 2.5 µm (PM_2.5_) is associated with obesity and type 2 diabetes (T2DM) development in genetically predisposed individuals [2], [42]. Many cohort studies reported a significant positive association between long-term PM_2.5_ exposure and T2DM, hypertension, and obesity in China, Canada, and South Korea [38], [46], [23], [28], [36], [34], [16], [44], [12], [13], [31]. Moreover, PM_2.5_ exposure induces low-grade inflammation and insulin resistance in rodents [40], [39], [58], [66]. A previous study from our group has shown that long-term exposure to PM_2.5_ increased low-grade inflammation in mice, leading to leptin resistance, hyperphagia, and decreased energy expenditure [10].

PM_2.5_ comprises diesel exhaust particles (DEP) with a complex mixture of solid, gaseous, and liquid fractions. Nitrogen oxides from diesel exhaust cause the nitration of carbon particles and form volatile organic compounds (VOCs) that may increase the toxicity and pro-inflammatory properties of these inhalable toxins [68]. In DEP, elemental carbon is the predominant component that absorbs other particles on its surface that, through oxidation, generates the chemical compound 1,2-naphthoquinone (1,2-NQ), which belongs to the quinones family [63], [25], [14], [56]. 1,2-NQ is present in significant concentrations in vehicle exhaust particles and ambient air samples, with approximately 13.7 μg of 1,2-NQ incorporated into each gram of DEP [14], [49].

1,2 NQ at high concentrations is an oxidizing agent, increasing oxidative stress and lipid peroxidation [9], [37], [52]. At low concentrations, 1,2-NQ significantly increases TNF-α protein levels in the alveolar space [17]. This response was associated with increased hydrogen peroxide (H2O2) [29], [51], [21]. 1,2-NQ may produce cellular damage via ROS, stimulating heat shock proteins (HSP) production, such as HSP70, which might act as a TLR4 agonist. The activation of TLR4 recruits MyD88 that subsequently stimulates the intracellular signaling cascade activating the NF-κB pathway and increasing pro-inflammatory cytokines such as TNF-α [59], [15], [26], [48], [49].

In obesity, chronic overfeeding can increase fat mass, causing a macrophage infiltration of adipose tissue, which results in low-grade systemic inflammation [30], [18]. The 1,2-NQ can initiate an inflammatory process in the lungs, delivering inflammatory mediators to peripheral tissues and priming obesity to settle.

Since 1,2-NQ production increases, comparable to diesel emissions, and has detrimental effects on immunity and metabolism, it is urgent to study how 1,2-NQ could affect health. Thus, we hypothesized that subchronic exposure to 1,2-NQ affects body weight, adiposity, food intake, and impaired glucose metabolism. Therefore, in the present study, we aimed to investigate whether a low concentration of subchronic exposure to 1,2-NQ may alter body mass and composition, food intake, and energy expenditure. Besides, we investigated whether subchronic exposure to 1,2-NQ may change glucose and insulin tolerance. Since 1,2-NQ is associated with alterations in the immune system, we also studied the contribution of 1,2-NQ exposure on the polarization of adipose tissue macrophages (ATMs) in the white adipose tissue (WAT) of WT mice and whether the tumor necrosis factor receptor 1 (TNFR1) and toll-like receptor 4 (TLR4) might be involved in this process.

## 2 Materials and methods

### 2.1 Origin, maintenance, and experimental design

The multidisciplinary Center for biological research (University of Campinas, SP, Brazil) and the University of São Paulo (USP) (Ribeirao Preto, SP, Brazil) provided all mice used in the study. The Animal Use Ethics Committee (CEUA 4628–1) and the Internal Biosafety Committee (CIBio protocol 2016/01) with Biosafety Quality Certification (CQB: 370) are in agreement with the guidelines of the Brazilian Council for Animal Experimentation (CONCEA), approved all the experiments displayed here.

We calculate the sample size using a formula suggested by the Animal Ethical Commission (CEUA/UNICAMP). The formula evaluates the Continuous Variables (Studies Comparing Two Group Means) [55], [19] considering the experimental groups and subgroups to obtain a test power of 90% and a significance level of 0.05. We used the following formula for the calculation base: *n = 1 + [2 C* **×** *(s/d)*^*2*^*]*, where: (C) depends on the values chosen for the power of the test; (s) is the acceptable standard deviation; (d) expected difference between groups. We used the following formula to calculate C: *C = (zα + zβ)*^*2*^, where: (z) statistical convention; (α) chance of wrongly considering two different groups; (β) chance of finding a statistical difference. For this study, we then obtained (C) = (1.96 + 1.282)^2^ = 10.51, considering the maximum deviation (s) of 20% = 0.2, and the difference between the groups studied (d) being 50% = 0.5. Thus, the sample size was n = 1 + [2 **×** 10.51 **×** (0.2/0.5)^2^] = 4.36, rounded to a minimum of 5 animals per experimental subgroup [55], [19].

All mice were male 8-week-old when started the treatment, including wild-type controls and deficient for TNFR1 or TLR4. The TLR4-deficient mice, named TLR4-/- and TLR4KO mice, were generated by homologous recombination in E14.1 in embryonic stem cells. These mice have a mutation on the third exon of the TLR4 gene. A substitution of C to A on the 2342 gene position changes proline to histidine on position 712 in the amino acids sequence [22]. This strain was validated and displayed a lack of response to LPS [22].

We also used TNFR1KO mice, originally called TNFR p55-deficient mice. These mice were generated by homologous recombination in C57BL/6-derived stem cells [45], in which a neomycin strand was inserted at position 535 of the coding sequence [45]. They were validated, displaying a selective lack of TNFR p55 and several deficits in inflammatory responses [45]. TLR4KO and TNFR1KO mice were healthy and have been employed in other studies [10], [27]. They were backcrossed on C57BL/6 genetic background and bred in the multidisciplinary center for biological research (University of Campinas, SP, Brazil) and the University of São Paulo, Ribeirão Preto, SP, Brazil. Here, we provided TNFR1 protein levels (sc-8436 mouse monoclonal) and TLR4 protein levels (sc-293072 mouse monoclonal) corrected by beta-actin (FINE TEST beta-actin Antibody_Fnab00869_rabbit polyclonal) from the pancreas of our transgenic and wild type mice (Supplemental Materials 1 A-B).

Supplemental Material 1 A. TNFR1 protein levels (sc-8436 mouse monoclonal) and B. TLR4 protein levels (sc-293072 mouse monoclonal) corrected by beta-actin (FINE TEST beta-actin Antibody_Fnab00869_rabbit polyclonal) from the pancreas of our transgenic and wild type mice.

All mice were housed in individual cages to allow the control of food intake. With controlled temperature (22–23 °C), fixed light and dark cycles (12 h/12 h), receiving a standard chow diet (3.39 kcal/g; Nuvilab CR-1, Nuvital Quimtia, Brazil), and filtered water ad libitum.

### 2.2 Body mass and food intake determination

We measured the body mass and food intake for five consecutive days before the initial exposures and considered the average as baseline data. After that, we exposed mice to 1,2-NQ or its vehicle for up to 17 weeks. During this time, we recorded body mass and food intake daily, and the average of the week was considered for calculation. For calculations of body mass evolution and food intake, we used the following formulas: **Body Mass -** Δ% = [(*final mass* - *initial mass)* / *initial mass*] **×** 100; **Food Intake -** Δ% = [(*final intake* - *initial intake)* / *initial intake*] **×** 100.

After 17 weeks of exposure to the 1,2-NQ or vehicle, we euthanized mice by decapitation and collected the tissues as described below.

### 2.3 Exposure to 1,2-NQ

The exposure protocol and aerosol concentrations inside the chamber were previously determined by Santos et al. [49]. In summary, four to five animals were placed in a polyethylene chamber with a surface area of 600 cm^2^, according to the dimensions proposed by the Canadian Manual of Animal Care [11]. The left side of the chamber had a 1.5 cm ventilation hole. The right side had a 2.0 cm polyethylene connector attached to the ultrasonic inhaler (Mod. Respiramax; NS®, São Paulo, Brazil). Mice were exposed to a low concentration of 1,2-NQ (10 μg/m^3^ inside the chamber), thus simulating a possible exposure in urban areas or the amount of 1,2-NQ present in vehicle exhaust particles that is of approximately 13.7 μg of 1,2-NQ incorporated in each gram of DEP or particulate matter (PM_2.5_ μm) [14], [49].

It is essential to state that according to the WHO Global air quality guidelines, the unhealthy level of PM_2.5_ is above 15 μg/m^3^ 24-hour mean (https://www.who.int/news-room/fact-sheets/detail/ambient-(outdoor)-air-quality-and-health).

We prepared fresh solutions for every exposure. Briefly, we diluted the 1,2-NQ (Sigma™, St. Louis, MO, USA) from the stock solution in a buffer containing PBS, 0.001% DMSO, and 0.001% Tween (vehicle) to reach the concentration used in the exposures of our study. To perform the exposures, we used an ultrasonic nebulizer coupled directly to a chamber; the concentration of 1,2-NQ inside the polluted chamber was calculated by a standard equation resulting in 152 ng of nebulized 1,2-NQ with 2–3% loss [49]. Animals were exposed to vehicle or 1,2-NQ at 2 pm daily for 15 min 5 times a week for 17 weeks. Thus, our exposures adopted the lowest concentration of 1,2-NQ (10 nM/mL) [49]. Our experiments had a 10 μg/m^3^ concentration inside the chamber.

As for the experimental subject, we divided as described [vehicle exposure [(WT, TNFR1KO, and TLR4KO; n = 5 each group) and 1,2-NQ exposure (WT n = 6, TNFR1KO n = 7 and TLR4KO n = 5)].

### 2.4 Energy expenditure, insulin, and glucose tolerance

We measured the energy expenditure, insulin, and glucose tolerance after 1,2-NQ exposure for 6–9 weeks. Energy expenditure was determined by oxygen consumption (VO_2_) and carbon dioxide production (VCO_2_), as well as respiratory exchange ratio (RER) by indirect calorimetry in the Oxylet M3 system; PanLab/Harvard Apparatus. The animals were acclimatized for 24 h before starting measurements. We applied the following equation to calculate energy expenditure: *Energy expenditure (kcal/h) = (3,85 + (1232* **×** *RER))* **×** *VO*_*2*_ **×** *1,44.*

We performed an insulin tolerance test (ITT) after 6-hour fasting, starting the experiment at 13:00 h. We collected blood from the tail tip to measure blood glucose by glucometer (basal time point). Then, we injected insulin via intraperitoneal (IP) (1.5 U/kg). We collected blood after 5, 10, 15, 20, 25, and 30 min to record blood glucose concentration. We performed a glucose tolerance test (GTT) after 12-hour fasting, starting the experiment at 08:00 h. We collected blood from the tail tip to measure fasting blood glucose by glucometer (basal time point). We then injected glucose IP (1 g/kg). We collected blood after 15, 30, 45, 60, 90, and 120 min to record blood glucose concentration.

### 2.5 Body composition

Body composition analysis was performed after 14 weeks of exposure to 1,2-NQ, by the Albira™ Si microPET-CT equipment, with a computed tomography (CT) module. The equipment obtained mice high-resolution anatomical images in a noninvasive quantitative manner, expressing the results in lean, fat, and bone masses. We kept the animals anesthetized with isoflurane during the experiment. The tomography lasted 35 min per animal. We used the Albira Suite software [50] for image acquisition.

### 2.6 Percentage of total macrophages, proinflammatory M1, and antiinflammatory M2 in epididymal white adipose tissue by flow cytometry

We euthanized the animals and dissected the epididymal adipose tissue at the end of the 17 weeks of exposure. Briefly, the epididymal adipose tissue was placed in a polypropylene Petri dish with 10 mL PBS (pH 7.4). We then macerated the tissue with a slide and placed it in the conical polypropylene tube (15 mL) containing 2 mL of white adipose tissue dissociation buffer (2.5%. HEPES, 10 mg/mL BSA, 3 mg/mL (0.3%) type II collagenase in DMEM with 4.5 g/L glucose without L-glutamine and sodium pyruvate). The samples were incubated with type II collagenase continuously at 37º C for 45 min. At the end of this process, the digested tissue was filtered into a conical polypropylene tube (50 mL) and then transferred to another smaller 15 mL tube with 2 mL DMEM. The cell suspension was centrifuged at 300g, 4 °C for 8 min. After centrifugation, floating adipocytes and supernatants were discarded, and the pellet was resuspended in 1 mL of potassium ammonium chloride buffer to remove erythrocyte (ACK buffer) and 1 mL of Dulbecco's modified eagle medium (DMEM) for another centrifugation at 300g, 4 °C for 8 min. Finally, the cells were resuspended in the 1x FACS buffer with PBS containing 2% fetal bovine serum (FBS), and live cells were counted in the Neubauer chamber [4]. For the BD Accuri™ C6 flow cytometer analysis, simglets gating was first applied, followed by F4/80 + gating strategy (Table 1 **and** Supplementary Material 2). A minimum number of 5000 F4/80 + macrophages (APC anti-mouse F4/80–1231116 - Biolegend) were counted and used for CD206 (FITC anti-mouse CD206 – 141703 – Biolegend) and CD11c (PE anti-mouse CD11c – 117307 – Biolegend) immunophenotyping. FMO (Fluorescence Minus One) controls were used for color compensation (Table 1 **and** Supplementary Material 2).

**Table 1:** Flow cytometer gate and control strategy for the analysis of the total number of macrophages (F4/80), M1 (CD11c), and M2 (CD206) from epididymal white adipose tissue. *FC Block prevented the binding of macrophage markers on CD16 and CD32. * *The fluorescence minus one (FMO) method was used to compensate for fluorophores and to adjust the fluorescence of each filter. * **Regarding the parameters for cytometer cell acquisition, 5000 events were determined within the positive F4/80 gate.

Control Type	Antibodies and Cells	Wavelength	Fluorescence	Filter
1- Unmarked	FC Block* + Macrophages * **	-	-	-
2- With 1 marking	FC Block* + Macrophages * ** + F4/80 APC (General)	640 nm	Red	675/25
3- With 1 marking	FC Block* + Macrophages * ** + CD11c PE (M1)	488 nm	Yellow	585/40
4- With 1 marking	FC Block* + Macrophages * ** + CD206 FITC (M2)	488 nm	Green	533/30
5- With 2 markings (FMO)* *	FC Block* + Macrophages * ** + F4/80 APC + CD11c PE	640 e 488 nm	Red and yellow	675/25 and 585/40
6- With 2 markings (FMO)* *	FC Block* + Macrophages * ** + F4/80 APC + CD206 FITC	640 e 488 nm	Red and green	675/25 and 533/30
7- With 2 markings (FMO)* *	FC Block* + Macrophages * ** + CD11c PE + CD206 FITC	488 e 488 nm	Yellow and green	585/40 and 533/30
8- Samples with 3 markings	FC Block* + Macrophages * ** + F4/80 APC + CD11c PE + CD206 FITC	640, 488 e 488 nm	Red, yellow and green	675/25, 585/40 and 533/30

### 2.7 Statistical analysis

We expressed the results as mean ± standard error of the mean (SEM) as absolute values or percentages indicated in the legends. For the analysis of two groups of mice, we used an unpaired two-tailed Student t-test. For comparing more than two groups of mice, we used the one-way analysis of variance (One-way-ANOVA) followed by the Tukey post-test. For comparing more than two groups of mice in addition to the time effect, we applied a two-way ANOVA test followed by the Bonferroni post-test. We also calculated the area under the curve for comparing more than two groups of mice in addition to the time effect. We used statistical software (GraphPad Prism 5.0®) to analyze all data. P < 0.05 were considered significant. All analyses were performed according to the software recommendation for each case.

## 3 Results

**Fig. 1:**
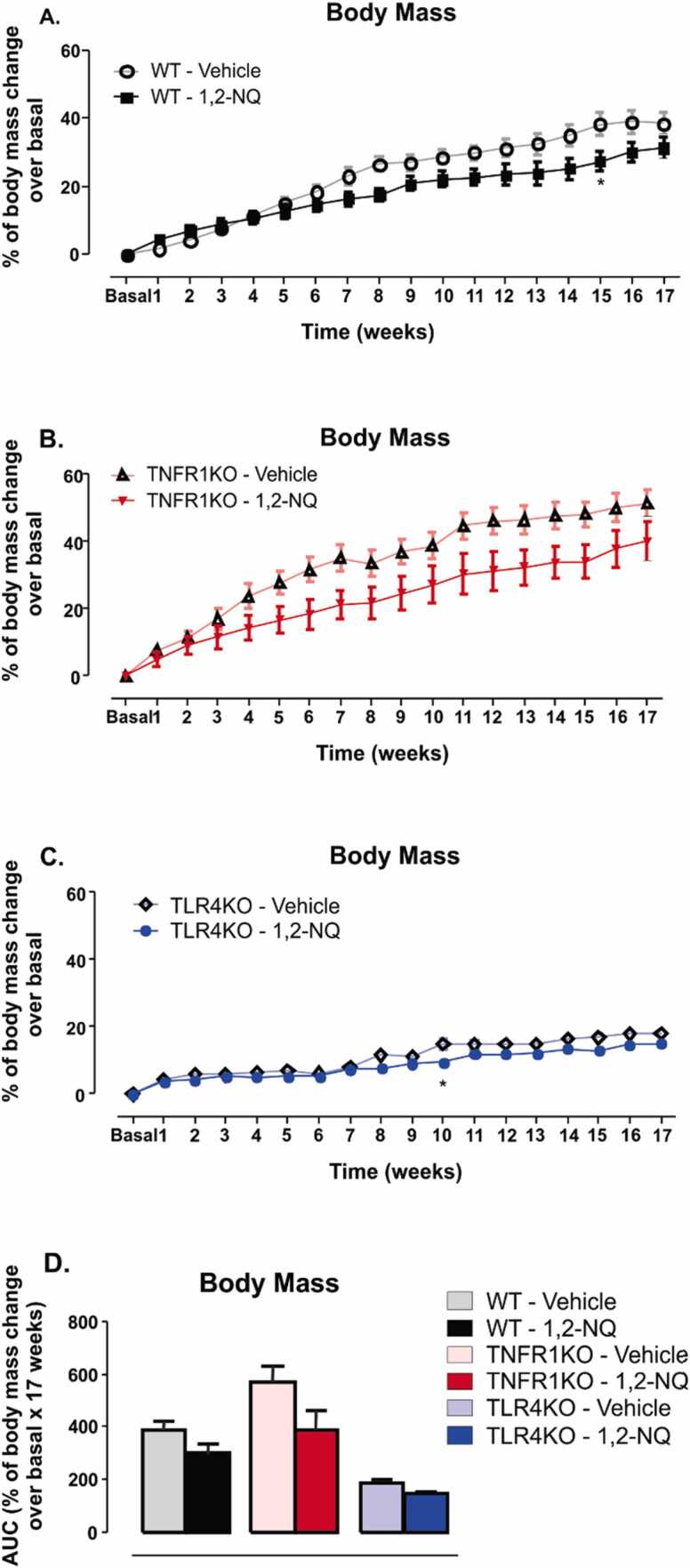
*Percentage of body mass change normalized to basal data during 17 weeks of 1,2-NQ or vehicle exposures.***(A-C)** Percentage of body mass change normalized to basal data; **(D)** The area under the curve (AUC). [WT, n = 5 (vehicle) and 6 (1,2-NQ); TNFR1KO, n = 5 (vehicle) and n = 7 (1,2-NQ); TLR4KO, n = 5 (vehicle) and 5 (1,2-NQ)]; Two-way ANOVA followed by Bonferroni post-test was applied for A-C; One-way ANOVA followed by Tukey post-test was applied for D. *p < 0.05 vs. vehicle. Values are expressed as the mean ± s.e.m.

There was a slight but not significant reduction in body mass of WT mice exposed to the 1,2-NQ compared to vehicle groups (Fig. 1 **A**). The area under the curve (AUC) analysis showed no statistical difference between the two groups (Fig. 1**D**). TNFR1KO mice exposed to 1,2-NQ showed a similar pattern of results to the vehicle group; however, no statistical difference was observed in any of the studied weeks (Fig. 1**B**), probably due to the large dispersion of the data. TLR4KO mice exposed to 1,2-NQ showed no difference in body mass in most f the weeks studied, except in the tenth week, where a significant reduction was observed (Fig. 1 **C**). The area under the curve was not statistically different among the groups (Fig. 1**D**). These results suggest that the exposure to 1,2-NQ did not affect body mass substantially in any mouse strains studied.

**Fig. 2:**
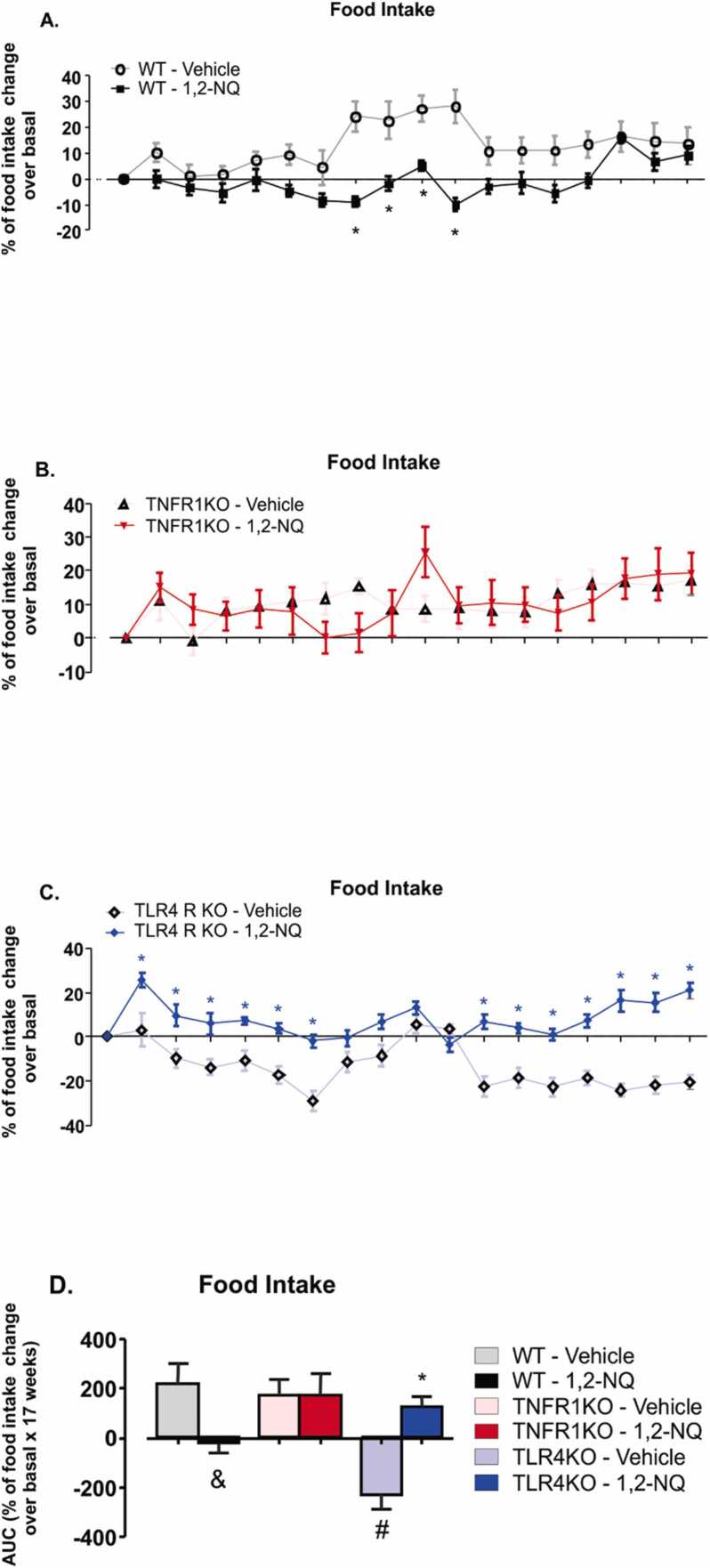
*Percentage of food intake change normalized to basal data during 17 weeks of 1,2-NQ or vehicle exposures.***(A-C)** Percentage of food intake change normalized to basal data; **(D)** The area under the curve (AUC). [WT, n = 5 (vehicle) and 6 (1,2-NQ); TNFR1KO, n = 5 (vehicle) and 7 (1,2-NQ); TLR4KO, n = 5 (vehicle) and 5 (1,2-NQ)]; Two-way ANOVA followed by Bonferroni post-test was applied for A-C; One-way ANOVA followed by Tukey post-test was applied for D. *p < 0.05 vs vehicle; #p < 0.05 vs WT undergoing the same treatment; &p < 0.05 two-tailed unpaired t-test in WT mice. Values are expressed as the mean ± s.e.m.

Food intake in WT mice exposed to the vehicle was higher from the seventh to the tenth week compared to its respective group exposed to 1,2-NQ (Fig. 2**A**). A statistically significant reduction was observed in the AUC data of the WT group exposed to the pollutant compared to the group exposed to the vehicle (Fig. 2**D**). TNFR1KO mice exposed to 1,2-NQ showed no reduction in food intake compared to the vehicle-exposed group (Fig. 2**B**), as in wild-type mice. On the other hand, TLR4KO exposed to 1,2-NQ had the highest food intake observed from the first to the sixth week and from the eleventh to the seventeenth week (Fig. 2**C**). In calculating the area under the curve, there was an increase in food intake in TLR4KO mice exposed to 1,2-NQ relative to the pollutant vehicle and a reduction in food intake in vehicle-exposed TLR4 compared to WT mice with corresponding exposure (Fig. 2**D**). These results suggest that exposure to 1,2-NQ affected food intake. 1,2-NQ exposure decreased food intake in WT mice, and the absence of TNFR1 prevented this effect. A lack of TLR4 increased food intake in response to 1,2-NQ.

**Fig. 3:**
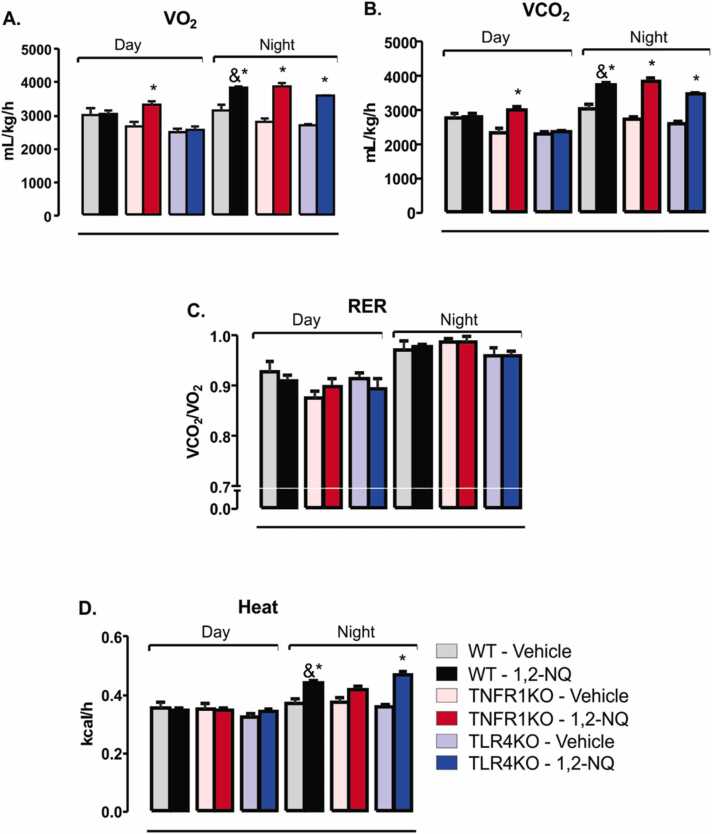
*Energy expenditure from mice exposed to 1,2-NQ or vehicle*. **(A)** Oxygen consumption (O2); **(B)** Carbon dioxide production (CO2); **(C)** respiratory exchange ratio (RER); **(D)** Heat. [WT, n = 5 (vehicle) and 4 (1,2-NQ); TNFR1KO, n = 5 (vehicle) and 5 (1,2-NQ); TLR4KO, n = 5 (vehicle) and 4 (1,2-NQ)]. One-way ANOVA followed by Tukey post-test was applied for A-D. *p < 0.05 vs vehicle; &p < 0.05 two-tailed unpaired t-test in WT mice. Measurements were done after 6 weeks of exposure. Values are expressed as the mean ± s.e.m.

In the sixth week of exposure to pollutant 1,2-NQ, we demonstrated that O_2_ consumption, CO_2_, and heat determined during the dark cycle increased in WT mice exposed to 1,2-NQ compared to vehicle-exposed mice (Fig. 3**A, B, and D**). TNFR1KO and TLR4KO exposed to 1,2-NQ had the same result pattern as their respective vehicle-exposed controls (Fig. 3**A, 3B, and 3D**). Only TNFR1KO animals exposed to 1,2-NQ showed no increase in heat during the dark cycle. They displayed an increase in O_2_ consumption and CO_2_ production compared to the vehicle during the light cycle compared to their respective control of the same genotype (Fig. 3**A and 3B**). In RER, there was no significant difference in any of the groups (Fig. 3**C**). These results suggest that 1,2-NQ exposure increased energy expenditure in the dark phase independently of the genotype.

**Fig. 4:**
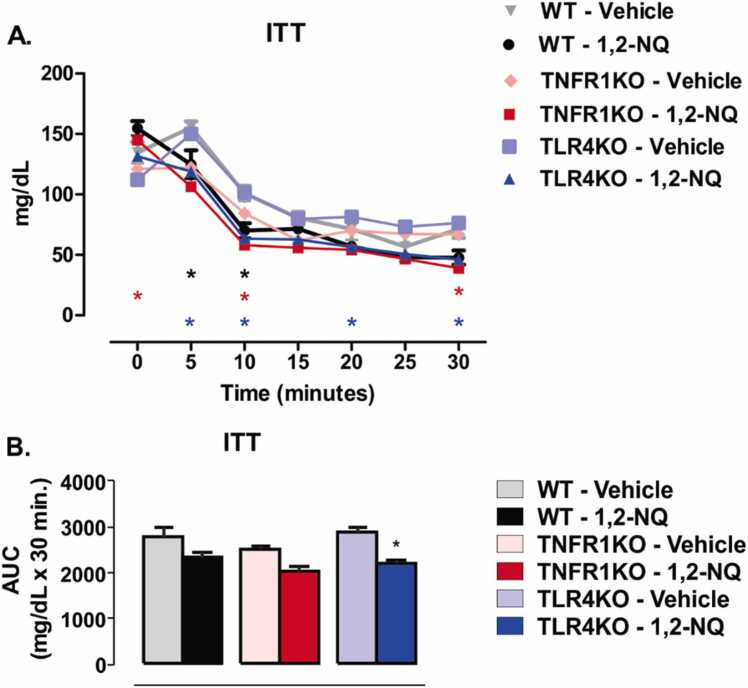
*Insulin tolerance test (ITT) from mice exposed to 1,2-NQ or vehicle*. **(A)** ITT curve [WT, n = 5 (vehicle) and 6 (1,2-NQ); TNFR1KO, n = 5 (vehicle) and 7 (1,2-NQ); TLR4KO, n = 5 (vehicle) and 5 (1,2-NQ)]; **(B)** The area under the curve (AUC); Two-way ANOVA followed by Bonferroni post-test was applied for A; One-way ANOVA followed by Tukey post-test was applied for B. *p < 0.05 vs. vehicle. Measurements were done after 8 weeks of exposure. Values are expressed as the mean ± s.e.m.

We performed the insulin tolerance test (ITT) after eight weeks of exposure to the pollutant 1,2-NQ or vehicle. TNFR1KO mice exposed to the 1,2-NQ displayed an increase in blood glucose than their respective vehicle before an insulin injection. Despite increased blood glucose at the beginning of the ITT, TNFR1KO displayed a significant decrease in blood glucose at 10 and 30 min after insulin injection compared to their respective vehicle (Fig. 4**A**). The WT mice exposed to 1,2-NQ displayed a reduction in blood glucose at 5 and 10 min compared to their vehicle-treated mice (Fig. 4**A**). TLR4KO mice exposed to the 1,2-NQ showed higher insulin tolerance, with reduced blood glucose in response to insulin during most test time points (Fig. 4**A**). In addition, the TLR4KO group exposed to 1,2-NQ had decreased AUC, indicating higher insulin sensitivity when compared to the vehicle of the same genotype (Fig. 4**B**). We did not observe significant differences in the AUC of WT and TNFR1KO mice exposed to the 1,2-NQ and their respective vehicle-treated groups (Fig. 4**B**). These results suggest a mild increase in insulin sensitivity in all mice exposed to 1,2-NQ.

**Fig. 5:**
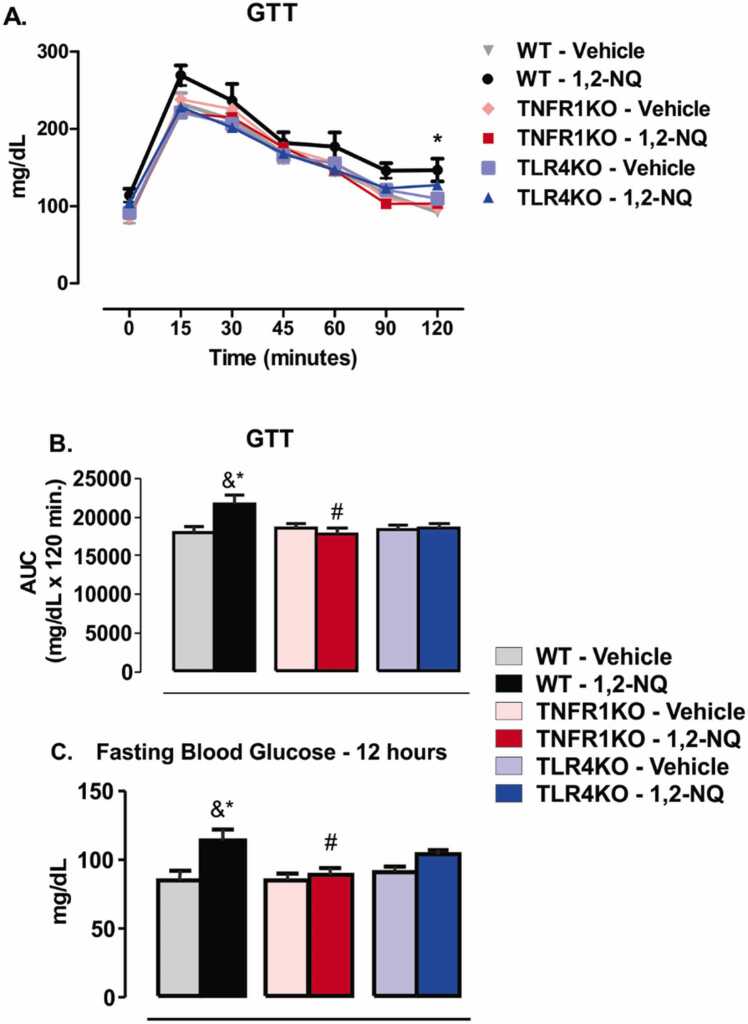
*Glucose tolerance test (GTT) from mice exposed to 1,2-NQ or vehicle*. **(A)** GTT curve [WT, n = 5 (vehicle) and 6 (1,2-NQ); TNFR1KO, n = 5 (vehicle) and 7 (1,2-NQ); TLR4KO, n = 5 (vehicle) and 5 (1,2-NQ)]; **(B)** The area under the curve (AUC); **(C)** 12-hour fasting blood glucose. Two-way ANOVA followed by Bonferroni post-test was applied for A; One-way ANOVA followed by Tukey post-test was applied for B-C. *p < 0.05 vs vehicle; #p < 0.05 vs WT undergoing the same treatment; &p < 0.05 two-tailed unpaired t-test in WT mice. Measurements were done after 9 weeks of exposure. Values are expressed as the mean ± s.e.m.

At week 9 of exposure, we observed increased blood glucose during the GTT in WT mice exposed to 1,2-NQ compared to vehicle-exposed mice (Fig. 5**A**). However, TNFR1KO and TLR4KO mice exposed to the 1,2-NQ did not display a difference in blood glucose during the GTT compared to their respective controls (Fig. 5**A**). The area under the GTT curve was increased in the 1,2-NQ WT group compared to the vehicle group suggesting that 1,2-NQ exposure is associated with glucose intolerance in the WT mice (Fig. 5**B**). 1,2-NQ TNFR1KO group displayed lower AUC than their vehicle group (Fig. 5**B**). There is no significant difference in the AUC between TLR4KO mice exposed to the 1,2-NQ or the vehicle (Fig. 5**B**). Therefore, the absence of TNFR1 and TLR4 partially protects from 1,2-NQ-induced glucose intolerance.

We observed a profound increase in fasting blood glucose in the 1,2-NQ WT group compared to the vehicle group (Fig. 5**C**). 1,2-NQ TNFR1KO group displayed an increase in fasting blood glucose than the vehicle group; however, in a mild manner (Fig. 5**C**). There is no significant difference in fasting blood glucose between TLR4KO mice exposed to the 1,2-NQ or the vehicle (Fig. 5**C**). These results suggest that subchronic exposure to 1,2-NQ increases fasting blood glucose and induces glucose intolerance in WT mice, but TNFR1 and TLR4 knockout mice remained partially protected from these effects.

**Fig. 6:**
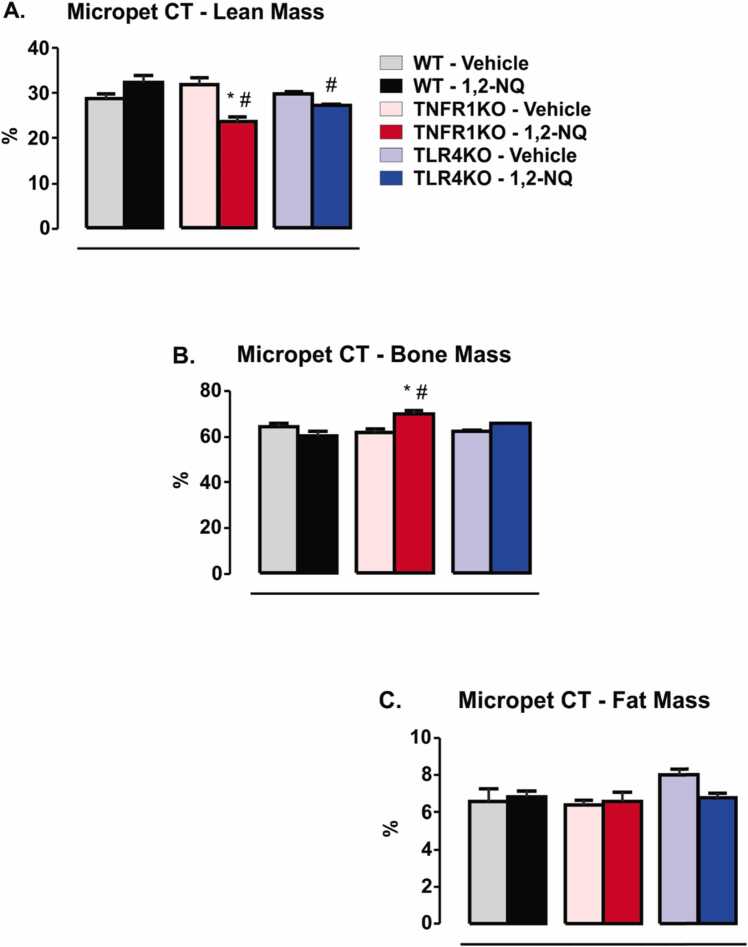
*Body composition by MicroPet-CT high-resolution computed tomography from mice exposed to 1,2-NQ or vehicle*. **(A)** Lean mass percentage; **(B)** Percentage of bone mass; **(C)** Fat mass percentage. [WT, n = 5 (vehicle) and 6 (1,2-NQ); TNFR1KO, n = 5 (vehicle) and 7 (1,2-NQ); TLR4KO, n = 5 (vehicle) and 5 (1,2-NQ)]. One-way ANOVA followed by Tukey post-test was applied for A-C. *p < 0.05 vs vehicle; #p < 0.05 vs WT undergoing the same treatment. Measurements were done after 14 weeks of exposure. Values are expressed as the mean ± s.e.m.

**Fig. 7:**
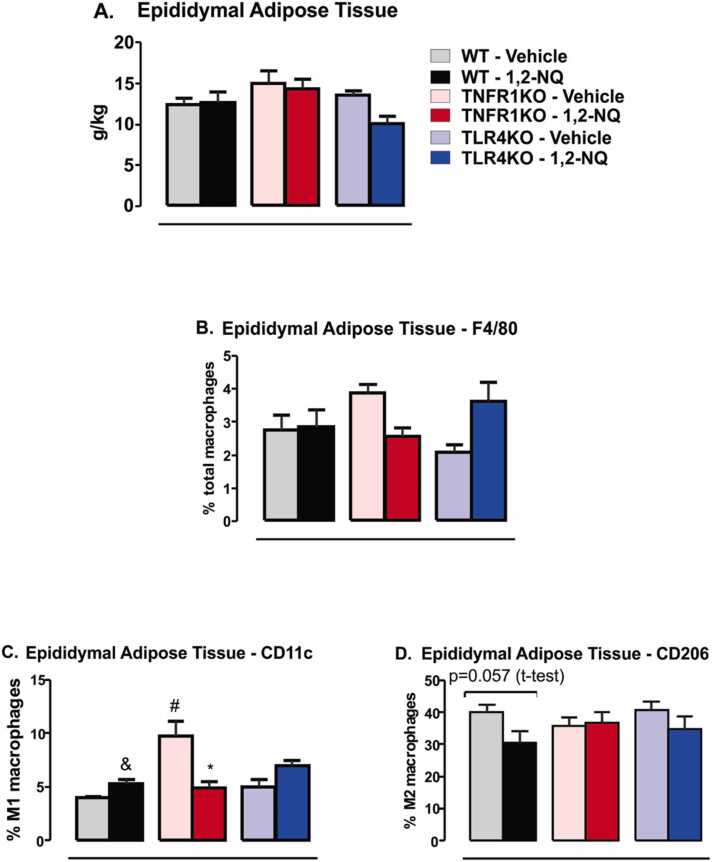
*Inflammatory profile of epididymal white adipose tissue from mice exposed to 1,2-NQ or vehicle*. **(A)** Epididymal fat tissue mass; **(B)** % of total F4/80 labeled macrophages; **(C)** % of CD11c-labeled M1 macrophages; **(D)** % CD206-labeled M2 macrophages. [WT, n = 5 (vehicle) and 6 (1,2-NQ); TNFR1KO, n = 5 (vehicle) and 7 (1,2-NQ); TLR4KO, n = 5 (vehicle) and 5 (1,2-NQ)]. One-way ANOVA followed by Tukey post-test was applied for A-D. *p < 0.05 vs vehicle; #p < 0.05 vs WT undergoing the same treatment; &p < 0.05 two-tailed unpaired t-test in WT mice. Measurements were done after 17 weeks of exposure. Values are expressed as the mean ± s.e.m.

We recorded the body composition after fourteen weeks of exposure. At this time point, we did not observe a statistical difference in the fat mass or lean and bone mass in WT exposed to 1,2-NQ compared to the vehicle-exposed WT mice (Fig. 6). In contrast, TNFR1KO mice exposed to 1,2-NQ demonstrated a reduction in lean mass and an increase in bone mass compared to vehicle-TNFR1KO mice (Fig. 6**A and 6B**). Comparing TNFR1KO and TLR4KO versus WT mice exposed to 1,2-NQ, we observed a decrease in the lean mass of TNFR1KO and TLR4KO (Fig. 6**A)** and an increase in bone mass in the TNFR1KO compared to WT mice (Fig. 6**A and 6B**). The exposure to the atmospheric chemical pollutant did not affect the fat mass of any group studied (Fig. 6**C and 7A**). The present study demonstrated that subchronic exposure to 1,2-NQ for 14 weeks did not affect fat mass in any studied mouse strains.

The epididymal adipose tissue was collected at the end of the exposures. We did not observe a significant change in the fat mass of WT mice exposed to 1,2-NQ and vehicle (Fig. 7**A**). The percentage of total macrophages labeled with F4/80 also did not change in the WT exposed to the pollutant relative to the vehicle (Fig. 7**B**). However, WT mice exposed to 1,2-NQ showed an increase in CD11c-labeled M1 pro-inflammatory and a slight decrease (p = 0.057) in CD206-labeled M2 anti-inflammatories macrophages in the white adipose tissue compared to WT group exposed to vehicle (Fig. 7**C, D**). The absence of TNFR1 or TLR4 may protect at least in part the increase in the percentage of M1 macrophages in the white adipose tissue of 1,2-NQ-exposed mice (Fig. 7**C**). Together, these results suggest that the absence of TNFR1 and TLR4 may protect animals from the increased percentage of pro-inflammatory M1 macrophages in the white adipose tissue when exposed to 1,2-NQ. There was a slight decrease in CD206-labeled M2 anti-inflammatory macrophages in the white adipose tissue of WT mice exposed to 1,2-NQ. This slight decrease was not seen in the other TNFR1KO and TLR4KO groups exposed to 1,2-NQ compared to their vehicle groups (Fig. 7**D**). These results suggest that despite no differences in the adipose tissue mass after the subchronic exposure to 1,2-NQ, the macrophage profile changes in WT mice adipose tissue, resulting in low-grade inflammation. The absence of TLR4 and TNFR1 avoided changes in the macrophage profile, protecting, at least in part, from these detrimental effects of the 1,2-NQ exposure.

## 4 Discussion

In the present study, subchronic exposure to 1,2-NQ in WT mice altered food intake and energy expenditure with a slight change in whole-body mass. Despite no differences in the adipose tissue mass after 1,2-NQ exposure, we observed an increase in the pro-inflammatory M1 macrophages (CD11c marker) in the adipose tissue of WT exposed to 1,2-NQ compared to vehicle-exposed WT mice. In parallel, M2 anti-inflammatory macrophages (CD206 marker) decreased in the adipose tissue of WT exposed to 1,2-NQ. Deletion of TNFR1 and TLR4 protected, at least in part, from these detrimental effects of the 1,2-NQ exposure.

Air pollution is one of the most harmful causes of imbalance in the energy homeostasis triad, composed of body mass, food intake, and energy expenditure [6], [53], [60]. However, it is unknown which toxic components in air pollution are causing the most detrimental effect on metabolism. Our study was designed to investigate the impact of a specific atmospheric chemical pollutant. We chose 1,2-naphthoquinone due to its in vivo harmful effect and escalating vehicle exhaust particles in ambient air.

Despite a slight decrease of body mass in the WT exposed to 1,2-NQ compared to vehicle WT mice, the exposure to 1,2-NQ decreased food intake. However, the absence of TNFR1 protected this effect, suggesting a possible role for TNF-α maintaining food intake in the context of subchronic exposure to 1,2-NQ. The ability of TNF-α to alter food ingestion was studied before. In some forms of cancers, anorexia and cachexia may be attributed partly to the release of TNF-α [54]. The administration of TNF antiserum partially reversed the tumor-induced reduction in rodent food intake [54]. Conversely, in obesity, higher concentrations of TNF-α are found in the blood and the CNS [5], [41]. In this scenario, the elevation of TNF-α increases food intake and weight gain [5], [41]. This effect is mainly mediated by leptin resistance since leptin is a potent anorexigenic hormone [5].

The absence of TLR4 reversed hypophagia in response to 1,2-NQ. TLR4KO mice exposed to 1,2-NQ did not decrease food intake, as did the WT mice. Conversely, TLR4KO mice exposed to 1,2-NQ consumed more chow diet than TLR4KO mice exposed to the vehicle. Another study of our group demonstrated that the energy intake of TLR4KO mice did not differ between the polluted and the air-filtered group, suggesting that the deletion of TLR4 protected the mice from hypophagia [10]. The deletion of TLR2 might lead to obesity even if mice received a chow diet [20]. The mechanism by which the deletion of TLR2 increases body weight may be related to changes in gut microbiota composition [20]. Another study showed that the exposure of C57BL/6 J mice to PM_2.5_ for 3 weeks altered the intestinal microbiota, mainly the beta diversity [43]. We did not evaluate the composition of gut microbiota after 1,2-NQ exposures. Therefore, 1,2-NQ exposure changing the gut microbiota of mice deserves further investigation and can contribute to clarifying the mechanism of body weight changes.

Our study observed that WT mice exposed to 1,2-NQ decreased food intake. The mechanism behind this effect is not yet clear. One possibility is that the 1,2-NQ exposure inhibits protein-tyrosine phosphatase 1B (PTP1B) action in vivo [1], [24]. PTP1B is a natural negative regulator of leptin signaling and is overexpressed in diet-induced obesity models [1], [57]. Neuronal PTP1B deletion in mice challenged with a high-fat diet ameliorates leptin action/signaling in the hypothalamus, contributing to decreased food intake and body weight [7]. However, further studies are necessary to determine whether hypothalamic PTP1B and leptin are involved in the hypophagia induced by 1,2-NQ exposure in WT mice.

In all groups, O2 consumption increased in response to 1,2-NQ exposure, inconsistent with body mass and food intake results. Notably, the measurement of O2 consumption was performed at the beginning of the exposures at 6 weeks of 1,2-NQ exposure. This effect reflects a more acute accurate impact of 1,2-NQ exposure since all mice strains showed increased O2 consumption. Even though body mass and food intake did not differ significantly between WT exposed to 1,2-NQ than vehicle exposed mice. Another explanation for increased nocturnal O_2_ consumption is the possibility that 1,2-NQ sub-products induce inflammation in the brown adipose tissue (BAT), impairing energy expenditure. In this sense, the mutagenesis of the nuclear transcription regulator Mecp2 (methyl CpG binding protein 2) in BAT-resident macrophages results in spontaneous obesity linked to altered energy expenditure, impairing thermogenesis. Mice lacking Mecp2 in CX3CR1 + macrophages in the BAT show low expression of thermogenic factors, such as UCP1, as a consequence of impaired sympathetic innervation with norepinephrine reduction [64]. As an inverse effect, in our study, there would be the possibility of a chronic increase of normal macrophages due to local inflammation in BAT, with a proportional rise in Mecp2, which could lead to increased energy expenditure.

In the present study, subchronic exposure to 1,2-NQ increases fasting blood glucose and induces glucose intolerance determined by GTT. Some effects on the liver might explain this phenotype. In this sense, some studies have shown that PM_2.5_ impairs hepatic functions compromising gluconeogenesis and impacting fasting blood glucose regulation [35], [47], [67]. Another possibility is that 1,2-NQ or a by-product reaches the pancreas causing local inflammation and impaired insulin production/secretion by pancreatic beta cells. Indeed, an elevation of PM_2.5_ may compromise pancreatic beta-cell function [2]. The reduction in insulin production and secretion by pancreatic beta cells increases glycemia in fasting or response to food, or even during GTT, which would be a possible explanation for the result found in the present study.

The improvement of insulin sensitivity after 1,2 NQ exposure cannot be a consequence of weight loss or differences in adipose mass. One speculation for increased insulin tolerance found in all 1,2-NQ-exposed groups can be the ability of 1,2-NQ to inhibit PTP1B in peripheral tissues [1]. In this sense, higher PTP1B expression in tissues impairs the ability of insulin to bind to its receptor, inducing insulin resistance and causing obesity and T2DM [57].

Conversely, the inhibition of PTP1B in diet-induced obesity animals potentially improved insulin resistance and normalized plasma glucose and insulin levels without inducing hypoglycemia [62]. The deletion of TNFR1 and TLR4 protected mice from higher fasting blood glucose and glucose intolerance, both observed in WT mice exposed to 1,2-NQ. This effect suggests the potential relevance of TNFR1 and TLR4 receptors in the process that led the WT mice to impaired glucose metabolism.

Pulmonary inflammation induced by exposure to air pollutants may cause cytokine migration to other tissues [8]. Exposure for 17 weeks to PM_2.5_ promoted macrophage infiltration into the visceral adipose tissue of WT mice [40]. In the present study, subchronic exposure to 1,2-NQ did not alter epididymal fat mass in WT mice.

On the other hand, there was an increase of M1 pro-inflammatory macrophages in this tissue accompanied by a slight decrease (p = 0.057) of M2 anti-inflammatory macrophages, suggesting the onset of low-grade inflammation. The deletion of TNFR1 and TLR4 was associated with no changes in adipose mass or M1 and M2% of total macrophages in adipose tissue. It is critical to mention that the reduction of M1 macrophages in TNFR1KO mice exposed to 1,2-NQ could reflect increased values of M1 macrophages in TNFR1KO mice exposed to the vehicle. It is unknown whether the 1,2-NQ or an intermediary product of 1,2-NQ can translocate from the alveoli into the bloodstream and directly change the macrophage profile in adipose tissue. Alternatively, both mechanisms may be involved in adipose tissue macrophage changes.

Overall the present study demonstrated that subchronic exposure to 1,2-NQ did not affect body mass or fat mass over time in any mouse strains studied.

An increase in weight and white adipose mass in WT mice was expected, which probably did not occur because mice were fed a standard balanced diet. On the contrary, food intake was cumulatively lower in WT mice exposed to 1,2-NQ. TNFR1KO mice were protected from reduced food intake, and TLR4KO had the opposite effect. Increased nocturnal energy expenditure and insulin tolerance were observed in all strains exposed to 1,2-NQ. On the other hand, WT exposed to 1,2-NQ displayed higher fasting blood glucose and glucose intolerance, but the TNFR1 and TLR4 knockout mice remained protected from these effects. Increased pro-inflammatory M1 macrophages and a slight decrease in M2 were observed in the epididymal adipose tissue from WT mice exposed to 1,2-NQ. These results suggest that subchronic exposure to 1,2-NQ changes the macrophage profile in adipose tissue before the onset of overweight and obesity. The absence of TLR4 and TNFR1 avoided changes in the macrophage profile.

In conclusion, our study suggests that subchronic exposure to 1,2-NQ in vivo is harmful, slightly reducing feeding and body mass, increasing inflammation in adipose tissue and fasting blood glucose, and impairing glucose tolerance. TNFR1 and TLR4 are partially involved in these outcomes.

## CRediT authorship contribution statement

**Clílton Kraüss de Oliveira Ferreira:** Term, Conceptualization, Methodology, Validation, Formal analysis, Investigation, Writing – original draft, Writing – review & editing, Visualization, Project administration. **Clara Machado Campolim:** Investigation, Writing – review & editing, Project administration. **Olívia Pizetta Zordão:** Methodology, Investigation. **Fernando Moreira Simabuco:** Methodology, Investigation. **Chadi Pellegrini Anaruma:** Formal analysis, Investigation. **Rodrigo Martins Pereira:** Formal analysis, Investigation. **Vitor Ferreira Boico:** Conceptualization, Investigation. **Luiz Guilherme Salvino:** Formal analysis, Investigation. **Maíra Maftoum Costa:** Formal analysis, Investigation. **Nathalia Quintero Ruiz:** Formal analysis, Investigation. **Leandro Pereira de Moura:** Resources, Supervision. **Mario Jose Abdalla Saad:** Resources, Funding acquisition. **Soraia Katia Pereira Costa:** Methodology, Writing – review & editing. **Young-Bum Kim:** Writing – review & editing, Supervision. **Patricia Oliveira Prada:** Conceptualization, Resources, Writing – review & editing, Supervision, Project administration, Funding acquisition.

## Declaration of Competing Interest

The authors declare that they have no known competing financial interests or personal relationships that could have appeared to influence the work reported in this paper.

## Data Availability

Data will be made available on request.
