# Supplementary figures and images for: Subchronic exposure to 1,2-naphthoquinone induces adipose tissue inflammation and changes the energy homeostasis of mice, partially due to TNFR1 and TLR4
Source: Toxicol Rep. 2023 Jun 15;11:10–22. doi: 10.1016/j.toxrep.2023.06.002 (PMC10293596; doi:10.1016/j.toxrep.2023.06.002)

# SUPPLEMENTARY MATERIAL

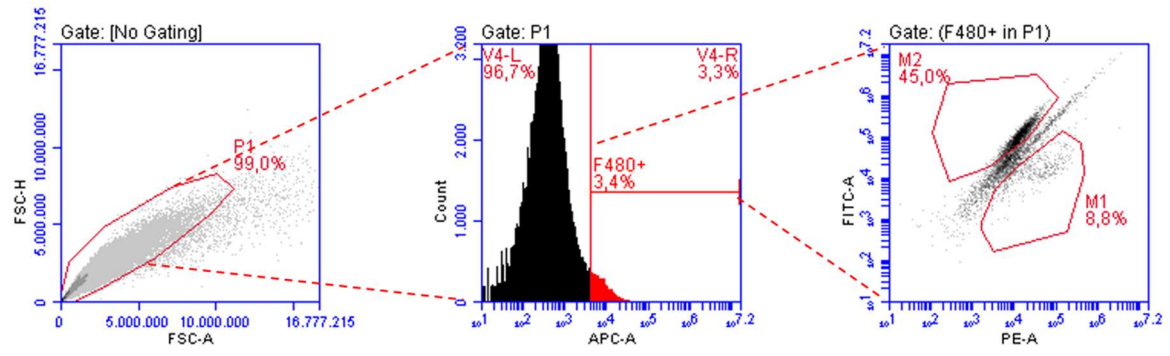

WT

TNF

TLR

C

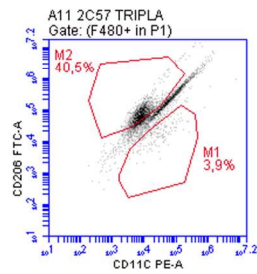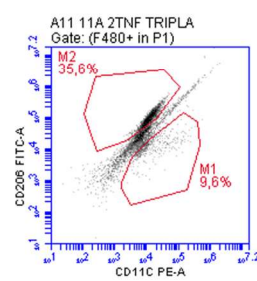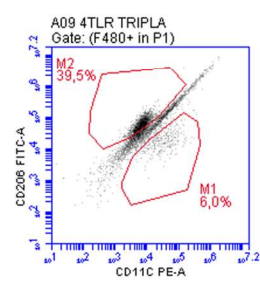

NQ

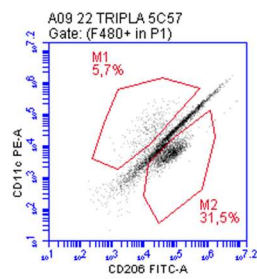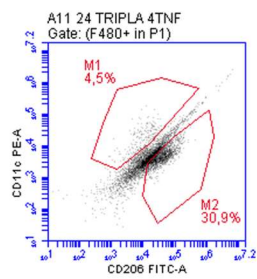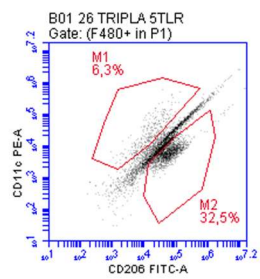

Supplement: Supplementary file 1 — Supplementary material. [file mmc1.pdf]

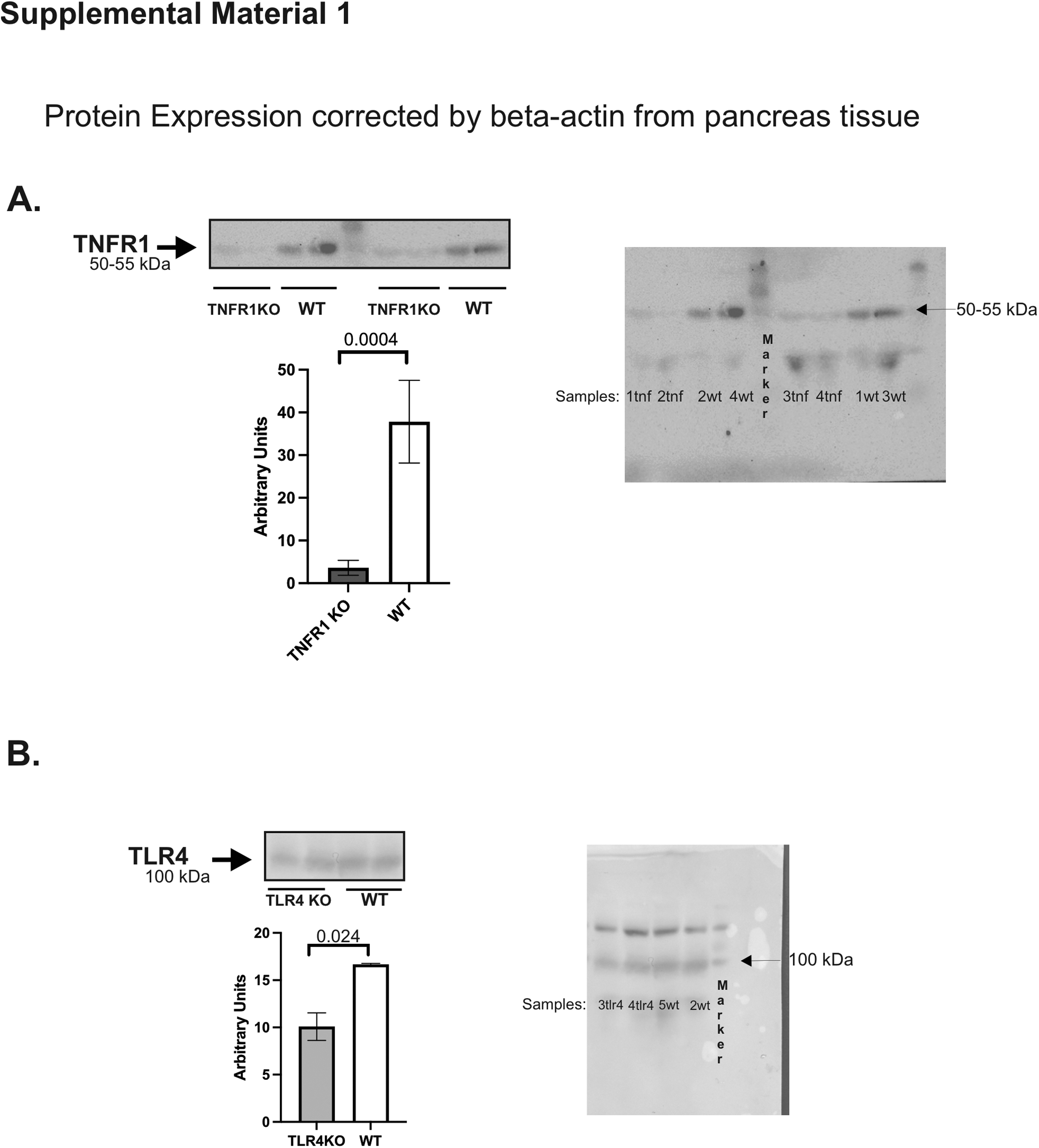

Supplement: Supplementary file 2 — Supplementary material. [file mmc2.jpg]
